# The Root of *Polygonum multiflorum* Thunb. Alleviates Non-Alcoholic Steatosis and Insulin Resistance in High Fat Diet-Fed Mice

**DOI:** 10.3390/nu12082353

**Published:** 2020-08-06

**Authors:** Soonwoong Jung, Hyeonwi Son, Chung Eun Hwang, Kye Man Cho, Sang Won Park, Hwajin Kim, Hyun Joon Kim

**Affiliations:** 1 Department of Anatomy and Convergence Medical Science, Bio Anti-Aging Medical Research Center, Institute of Health Sciences, Gyeongsang National University School of Medicine, Jinju 52727, Korea; birth1110@gnu.ac.kr (S.J.); haeny@gnu.ac.kr (H.S.); 2 Department of Food Science, Gyeongnam National University of Science and Technology, Jinju 52725, Korea; poponot@naver.com (C.E.H.); kmcho@gntech.ac.kr (K.M.C.); 3 Department of Pharmacology and Convergence Medical Science, Bio Anti-aging Medical Research Center, Institute of Health Sciences, Gyeongsang National University School of Medicine, Jinju 52727, Korea; parksw@gnu.ac.kr

**Keywords:** high-fat diet, insulin resistance, lipid, non-alcoholic steatosis, *Polygonum multiflorum*

## Abstract

Non-alcoholic steatosis and insulin resistance are critical health problems and cause metabolic complications worldwide. In this study, we investigated the molecular mechanism of *Polygonum multiflorum* Thunb. (PM) against hepatic lipid accumulation and insulin resistance by using in vitro and in vivo models. PM extract significantly attenuated the accumulation of lipid droplets and hepatic triglyceride in free fatty acid (FFA)-exposed HepG2 cells. PM extract increased the AMPK and ACC phosphorylation and GLUT4 expression, whose levels were downregulated in FFA-exposed cells. PM extract also decreased precursor and mature forms of SREBP-1 in FFA-exposed cells. C57BL/6 mice fed with normal diet (ND) or high-fat diet (HFD) were administered PM extract (100 mg/kg) or vehicle orally for 16 weeks. PM extract attenuated the increases of the epididymal and perirenal fats on HFD feeding. PM extract markedly reduced hepatic lipid accumulation and fasting glucose levels, and improved glucose and insulin sensitivity in HFD-fed mice. HFD-fed mice decreased the AMPK and ACC phosphorylation and GLUT4 expression, and increased precursor and mature forms of SREBP-1; these changes were significantly restored by PM extract. In conclusion, PM extract alleviates non-alcoholic steatosis and insulin resistance through modulating the expression of proteins on lipid metabolism and glucose transport in the liver.

## 1. Introduction

Non-alcoholic fatty liver disease (NAFLD) encompasses from simple steatosis to nonalcoholic steatohepatitis (NASH), advanced fibrosis or cirrhosis and hepatocellular carcinoma, and is a rising health concern linked to the development of numerous health problems and high costs. NAFLD is found in 25–35% and 5–15% of the general population of Western and Asian countries, respectively [1]. NAFLD is closely related to obesity and dyslipidemia, which ultimately contributes to insulin resistance, type 2 diabetes, obstructive sleep apnea, and cancer [2,3]. The incidence of NAFLD is higher in people with type 2 diabetes (60–70%), and in those who are obese or morbidly obese (75–92%), compared to the general population [4]. NAFLD is continuously increasing worldwide with the prevalence of obesity and diabetes; however, the pathogenesis and underlying molecular mechanism of NAFLD progression is yet to be revealed.

The non-alcoholic fatty liver (simple steatosis), the early stage of NAFLD, is accompanied by excessive lipid or triglyceride (TG) deposition in hepatocyte without significant alcohol intake or the initiation of inflammation. As the liver is a central organ regulating lipid homeostasis through lipogenic and lipolytic balance, the disruption of theses metabolic pathways may precipitate the hepatic retention of fat. Hepatic TG content is obtained 60% from free fatty acids (FFA) of adipose tissues, 26% from de novo lipogenesis, and 15% from the diet in steatotic patients [5]. However, in healthy individuals, de novo lipogenesis contributes to only < 5% of their hepatic TG [6]. Non-alcoholic steatosis is considered to be an optimal therapeutic stage of NAFLD since it is reversible to normal condition by restoring the metabolic homeostasis.

Obesity is developed by a sedentary life style, inactivity, and over-eating, and the obese individuals often exhibit altered lipid metabolism [7], where non-alcoholic fatty liver is very common. The elevated FFA levels and TG accumulation are important causes of obesity-induced insulin resistance; however, the underlying mechanisms are not completely known. Lipid metabolites, pro-inflammatory cytokines, and oxidative or ER stress have shown to be involved, and their intervention has been currently investigated to alleviate the FFA-mediated insulin resistance [8].

Adenosine monophosphate activated kinase (AMPK) is a highly conserved sensor of cellular energy deletion and stresses, and rapidly activated targeting towards increased catabolism and decreased anabolism [9]. In particular, the activated AMPK inhibits fat synthesis by inactivating acetyl-CoA carboxylase (ACC) and 3-hydroxy-3-methylglutaryl-CoA reductase (HMG-CoA reductase, HMGR), rate-limiting enzymes of the lipogenic pathways. AMPK activation has been shown to inhibit SREBP1 activity to attenuate hepatic steatosis and atherosclerosis in diet-induced insulin-resistance mice [10]. AMPK also mediates changes in glucose homeostasis through regulating expression and translocation of glucose transporter 4 (GLUT4) [11,12,13]. In addition, AMPK activation also stimulates hepatic fatty acid oxidation and inhibition of adipocyte lipogenesis, all of which suggests that AMPK is an attractive target for managing hepatic metabolic disorders and insulin resistance [14].

*Polygonum multiflorum* Thunb. (PM) is one of the most popular Chinese traditional medicines known as He shou wu in China and East Asia. It is considered as one of the top three Chinese medicines along with *Lycium chinense* and *Panax ginseng*. PM has been widely used for the treatment of various diseases such as liver injury, cancer, diabetes, atherosclerosis, and neurodegenerative diseases as well [15]. However, due to the toxicity of raw PM, the processing procedures are required to reduce the toxic ingredients such as emodin and its derivatives [16]. Traditionally, PM was processed as nine cycles of steaming and solarization, or was prepared as an ethanol fermentation method. In this study, PM extract was prepared by ethanol extraction, filtration, evaporation, and dissolution by sonication through multiple rounds of optimization. Then, we investigated the effect of PM extract at a non-cytotoxic concentration to determine whether it could reduce the hepatic lipid accumulation and insulin resistance, and we further explore its underlying molecular mechanisms using in vitro and in vivo models.

## 2. Materials and Methods

### 2.1. Preparation of PM Extract

The processed PM powder was purchased from Jirisan-hasuo Farming Corporation (Sancheong, Korea), and the PM extract was prepared by Kye Man Cho using an ethanol extraction method. The processing steps included repeated (usually 3–5) cycles of steaming and soaking of PM in the boiled water of Rhynchosia volubilis (jwinuni kong) and Korean rice wine. Ten g of dried PM was mixed with 200 mL of 70% ethanol, and was extracted by centrifugation at 600 rpm, 40 °C for 4 h. The extraction was repeated two times, and the three extracts were combined and filtered using filter papers (No. 2, Whatman, Tokyo Roshi Kaisha, Ltd., Tokyo, Japan). The filtered extract was concentrated at 60°C using a rotary evaporator (EYELA, Tokyo, Rikakikai Co., Tokyo, Japan). PM extract was dissolved in the water including 0.5% of Tween-20, and it was sonicated for 1 h. The final stock solution of PM extract was at 50 mg/mL. The extraction setting was optimized by a preliminary study, which determined the amount of phenolic compounds and flavonoids in the processes.

### 2.2. High-Performance Liquid Chromatography (HPLC)

The analyses of catechin, 2,3,4,5ʹ-tetrahydroxystilbene-2-O-α-glucoside (TSG), rhein, emodin, and chrysophenol were performed on an Agilent 1200 Infinity series (Agilent Technologies, Santa Clara, CA, USA). Samples were separated on an XTerra RP18 analytical column (4.6 × 250 mm, 5 μm; Waters Corp., Milford, MA, USA). The mobile phase contained 0.5% glacial acetic acid in water (solution A) and 0.5% glacial acetic acid in acetonitrile (solution B). The injection volume was 20 μL, and the flow rate was 1.0 mL/min at 30 °C. The detection wavelength was 280 nm.

### 2.3. Animals and Treatments

Male C57BL/6 mice (3-week old) were purchased from KOATECH (Pyeongtaek, Korea) and maintained in the animal facility at Gyeongsang National University. The experiments were performed in accordance with the National Institutes of Health Guidelines on the Use of Laboratory Animals. The university animal care committee for animal research of Gyeongsang National University approved the study protocol (GNU-161004-M0055). Mice were housed with 12-h light/dark cycle at 25 °C and were allowed free access to water and normal diet for 1 week, before dividing into four experimental groups (10–11 mice per group). The mice fed with normal diet (ND; fat 6% of total kcal) or high-fat diet (HFD; fat 45% of total kcal) were administered PM (100 mg/kg) or vehicle (water) orally for 16 weeks. Mice were weighed twice in a week throughout the period of experiment. The mice were sacrificed after 16 weeks, and the liver and adipose tissues were weighed.

### 2.4. Cell Culture and Treatments

Human hepatoma HepG2 cells were purchased from the American Type Culture Collection (Manassas, VA, USA) and maintained in low-glucose Dulbecco’s modified Eagle’s medium (Invitrogen, Grand Island, NY, USA) supplemented with 10% (*v/v*) heat-inactivated FBS, penicillin G (100 U/mL), streptomycin (100 mg/mL), and L-glutamine (2 mM) at 37 °C in 5% CO_2_. After reaching 75% confluence, the cells were starved for 16 h, and then exposed to an FFA mixture to induce fat overloading. An FFA mixture at a 2:1 ratio of oleate/palmitate was diluted in a culture medium containing 1% fatty acid-free bovine serum albumin (BSA) to reach the desired final concentrations [17]. The control cells were treated with 1% fatty acid-free BSA.

### 2.5. Cytotoxicity of PM

We measured cell viability by two different methods using 3-(4,5-dimethylthiazol-2-yl)-2,5- diphenyltetrazolium bromide (MTT) and Real time cell analyzer (RTCA, Roche, Basel, Switzerland). The MTT assay was performed 24 h after PM treatment. Briefly, 1 mg/mL MTT solution was added to each well and incubated at 37 °C in 5% CO_2_ for 3 h. The medium was removed, and DMSO was added to dissolve the MTT-formazan complex. The absorbance was measured at 570 nm using a microplate reader (Infinite F200, Tecan Group Ltd., Männedorf, Switzerland). Cells were seeded at a density of 5000 cells/well in E-plate 16 (ACEA Biosciences, San Diego, CA, USA), and were monitored every 15 min in RTCA (Roche). When the cells reached the logarithmic growth phase, PM was treated at the concentrations of 0, 0.05, 0.1, or 0.2 mg/mL in quadruplicate, and cell viability was monitored by continuous impedance recording every 15 min in RTCA (Roche).

### 2.6. Measurement of Blood Glucose Levels

Fasting blood glucose levels of mice were measured after a 16 h (overnight) fasting every two weeks. A drop of blood was taken from the tail vein, and blood glucose levels were measured using an Optium Xceed glucometer (Abbott, Abbott Park, IL, USA).

### 2.7. Glucose Tolerance Test and Insulin Tolerance Test

Mice were fasted for 16 h before the glucose tolerance test (GTT). D-glucose (2 g/kg) was intraperitoneally injected, and blood samples were taken from the tail vein before and 30, 60, 90, and 120 min after the injection of glucose. Blood glucose levels were measured by a glucometer (Abbot). To perform the insulin tolerance test (ITT), mice were injected with 0.1 mL of 0.9% normal saline containing insulin (1 U/kg, Humulin-R; Eli Lilly and Company, Indianapolis, IN, USA). A drop of blood was taken from the tail vein before and 30, 60, 90, and 120 min after the injection of insulin, and blood glucose levels was measured by a glucometer (Abbot).

### 2.8. Triglyceride Levels in Liver Tissues and HepG2 Cells

Triglyceride levels in the liver tissues and HepG2 cells were measured using a commercially available TG Colorimetric Assay kit (Cayman, Ann Arbor, MI, USA) according to the manufacturer’s protocol. At 24 h after the treatment of FFA mixture, HepG2 cells were collected by centrifugation at 1000× *g* for 10 min at 4 °C.

### 2.9. H&E and Oil Red O Staining of Liver Tissues

The mice were perfused with 4% paraformaldehyde, and the liver tissues were embedded in paraffin for histological examination. Five m of paraffin liver sections were stained with H&E by standard methods [18]. Ten m of frozen liver sections were prepared, stained with 0.3% Oil-red O in 60% isopropanol for 40 min, and then were counterstained with hematoxylin. HepG2 cells were washed, fixed with 10% PBS-buffered formalin for 1 h at room temperature, and then stained with 2.1 mg/mL Oil-red O in 60% isopropanol for 10 min. The images were obtained under a BX51 light microscopy (Olympus, Tokyo, Japan). Oil-red O was eluted by adding 100% isopropanol, and then the quantity was measured at 545 nm using a microplate reader (Infinite F200).

### 2.10. Western Blot Analysis

Cells or liver tissues were homogenized in a lysis buffer and incubated on ice for 20 min. The supernatant was collected by centrifuging at 14,000× *g* at 4 °C for 10 min. Protein samples were separated on sodium dodecyl sulfate–polyacrylamide gel electrophoresis (SDS-PAGE), and transferred to polyvinylidene fluoride (PVDF) membranes (Roche). The membranes were incubated to the primary antibodies for adenosine monophosphate activated kinase (AMPK), phospho-AMPK, acetyl-CoA carboxylase (ACC), phospho-ACC (Cell Signaling Technology, Beverly, MA, USA), glucose transporter 4 (GLUT4) (Abcam, Cambridge, UK), sterol regulatory element-binding protein-1 (SREBP-1), -tubulin (Santa Cruz, Santa Cruz, CA, USA), and -actin (Sigma-Aldrich, Burlington, MA, USA), and then to respective secondary antibodies. The bound antibodies were detected using an ECL detection system (Pierce, Rockford, IL, USA) according to the manufacturer’s instructions. The Multi-Gauge version 3.0 image analysis program (Fujifilm, Tokyo, Japan) was used to measure the band densitometry.

### 2.11. Statistical Analysis

Statistical difference among the groups was determined with one-way analysis of variance (ANOVA), followed by Bonferroni post hoc analysis. The values are expressed as the mean ± SEM. A *p*-value < 0.05 was considered statistically significant.

## 3. Results

### 3.1. Phytochemical Contents in the PM Extract

We determined the contents of phytochemicals in PM extracts by HPLC analysis. Among the phytochemicals analyzed, 2,3,5,4ʹ-tetrahydroxystilbene-2-O-α-glucoside (TSG) was the most abundant, followed by catechin. The contents of rhein and emodin were low, but detectable; however, chrysophenol was not detected in our analysis (Table 1).

### 3.2. Cytotoxicity of PM in HepG2 Cells

**Figure 1:**
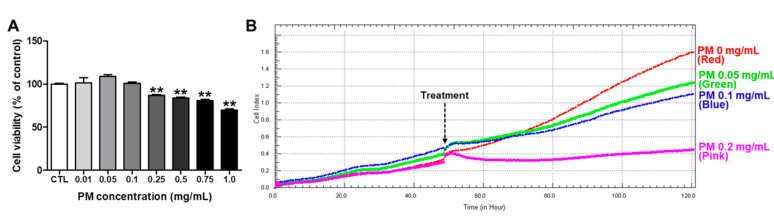
Cytotoxicity of PM in HepG2 cells. (**A**) HepG2 cells were treated with PM at the indicated concentrations for 24 h, and cell viability was measured by MTT assay. Data are the mean ± SEM from three independent experiments. ** *p* < 0.01 vs. CTL (control group). (**B**) The cells were seeded in E-plate 16, and proliferation, migration, and adherence were monitored every 15 min in the real time cell analyzer (RTCA) for 48 h. When the cells reached the logarithmic growth phase, PM was treated at the concentrations of 0, 0.05, 0.1, or 0.2 mg/mL in quadruplicate, and cell viability was monitored by continuous impedance recording every 15 min in RTCA.

We then determined a non-cytotoxic concentration of PM extract in HepG2 for following in vitro experiments by performing two different assays, MTT and RTCA. As shown in Figure 1, PM treatment did not reduce the cell viability significantly up to 0.1 mg/mL concentration in both MTT and RTCA.

### 3.3. PM Extract Attenuated the Increases of Lipid Accumulation and Intracellular TG Levels in FFA-Exposed HepG2 Cells

**Figure 2:**
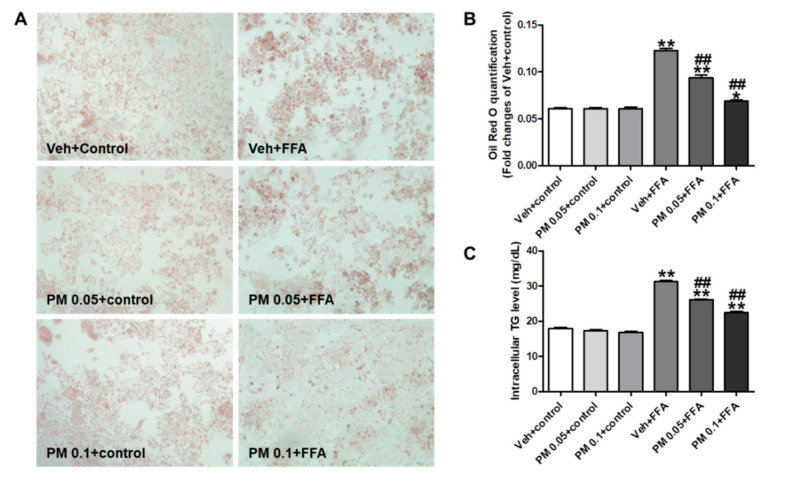
The effect of PM on intracellular lipid and triglyceride (TG) accumulation in free fatty acid (FFA)-treated HepG2 cells. Representative pictures (magnification 200X) of Oil-red O stain in HepG2 cells from three independent experiments (**A**), the quantification of lipid contents (**B**), and intracellular TG levels (**C**) were shown. Control groups were treated with 1% of fatty acid-free bovine serum albumin (BSA), and FFA groups were treated with 1 mM of FFA for 24 h, and then were harvested. PM was pre-treated 1 h prior to FFA at the indicated concentrations. Data are the mean ± SEM. ** *p* < 0.01 vs. Veh + control group; ^##^ *p* < 0.01 vs. Veh + FFA group.

To determine whether PM affects the hepatic accumulation of lipid, HepG2 cells were treated with 1 mM of FFA mixture for 24 h to induce the lipid accumulation. PM extract was pretreated at concentrations of 0.05 and 0.1 mg/mL prior to 1 h of FFA treatment, which are non-cytotoxic concentrations (Figure 2A). For FFA-exposed cells, the Veh + FFA group showed a 2.0-fold increase of lipid contents compared to the Veh + control group. However, the increase was attenuated by PM extract in a concentration-dependent manner (Figure 2B). Similar to the lipid accumulation, intracellular TG levels were significantly increased by FFA exposure, but the increase was attenuated by PM treatment in a concentration-dependent manner (Figure 2C). The treatment of PM did not affect the lipid contents or intracellular TG levels in control cells that were treated with 1% of fatty acid-free BSA.

### 3.4. PM Extract Modulated Lipogenic and Lipolytic Protein Levels in FFA-Exposed HepG2 Cells

**Figure 3:**
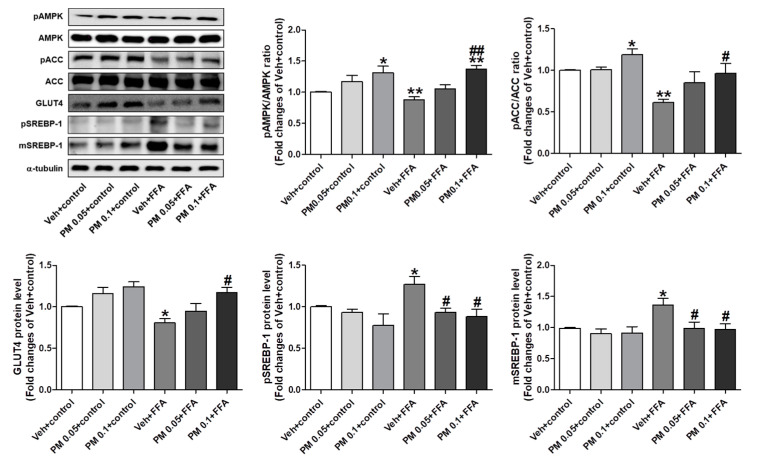
The effect of PM extract on the adenosine monophosphate-activated kinase (AMPK) and acetyl-CoA carboxylase (ACC) phosphorylation, glucose transporter 4 (GLUT4), and sterol regulatory element-binding protein-1 (SREBP-1) protein levels in FFA-treated HepG2 cells. Representative images of western blot analysis were from three independent experiments, and the densitometric quantification of relative band intensities are presented. The cells were treated with 1 mM of FFA for 24 h, and then were harvested. PM was pretreated 1 h prior to FFA treatment at the indicated concentrations, and control cells were treated with 1% of fatty acid-free BSA. Data are the mean ± SEM. * *p* < 0.05 and ** *p* < 0.01 vs. Veh + control group; ^#^ *p* < 0.05 and ^##^ *p* < 0.01 vs. Veh + FFA group.

Due to the effect of PM on limiting lipid accumulation, we further investigated whether PM extract affects the expression of proteins regulating lipolysis (AMPK and ACC) and lipogenesis (SREBP), as well as GLUT 4 in HepG2 cells. Total AMPK and ACC protein levels were not altered by FFA or PM extract. FFA exposure reduced the phosphorylation of AMPK and ACC, and the expression of GLUT4 protein; however, the reduced levels were significantly recovered by PM treatment at 0.1 mg/mL. Also, the treatment of PM alone significantly induced the phosphorylation of AMPK and ACC in control cells. SREBP has been known to be expressed as a precursor form, and has been cleaved to mature form to be translocated into the nucleus as an active transcription factor. Thus, we examined the protein levels of both precursor (p) and mature (m) forms of SREBP. FFA exposure significantly increased pSREBP and mSREBP levels, which were reduced by PM treatment (Figure 3).

### 3.5. PM Extract Attenuated the Weight Increase of Adipose Tissues without Changing Body Weight or Food Intake in HFD-Fed Mice

**Figure 4:**
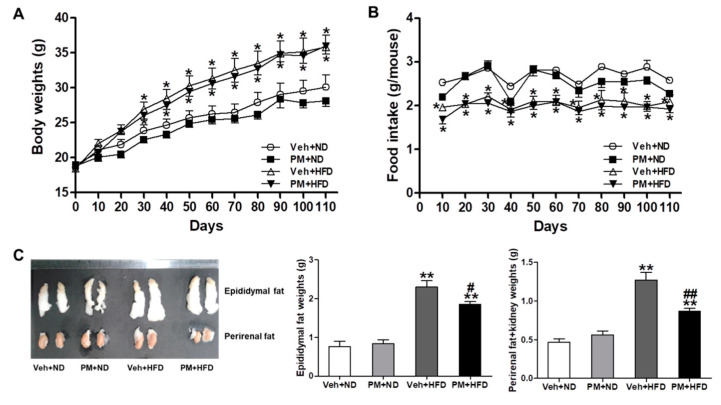
The effect of PM extract on body weight, food intake, and weight of adipose tissues in normal diet (ND)- or high-fat diet (HFD)-fed mice. The mice were administered PM extract or vehicle orally for 16 weeks, and the adipose tissues were collected. Body weight (**A**) and food intake (**B**) were measured every 10 days for 16 weeks. At sacrifice, representative images of epididymal and perirenal fats were taken, and the weight of these fats were measured. Data are the mean ± SEM. * *p* < 0.05 and ** *p* < 0.01 vs. Veh + ND group; ^#^ *p* < 0.05 and ^##^ *p* < 0.01 vs. Veh + HFD group.

Mice were fed with ND or HFD (45% in lipid composition), and were administered PM extract (100 mg/kg) or vehicle orally for 16 weeks. HFD-fed mice showed a significant increase of body weight and a decrease of food intake, compared to ND-fed mice; however, these changes were not affected by PM treatment (Figure 4A,B). After 16 weeks, the weight of adipose tissues was measured in ND- or HFD-fed mice. HFD-fed mice showed a significant increase of epididymal and perirenal fats, compared to ND-fed mice, but these increases were attenuated by PM extract (Figure 4C).

### 3.6. PM Extract Attenuated the Increases of Hepatic TG Levels and Hepatocellular Lipid Accumulation in HFD-Fed Mice

**Figure 5:**
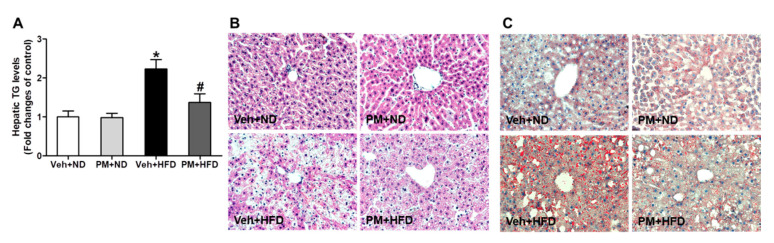
The effect of PM extract on hepatic triglyceride (TG) and lipid accumulation in HFD-fed mice. Hepatic TG levels (**A**) and representative images (magnification 200X) of H&E (**B**) and Oil-red O staining (**C**). The mice fed with ND or HFD were treated with PM extract or vehicle for 16 weeks, and the liver tissues were collected. Data are the mean ± SEM. * *p* < 0.05 vs. Veh + ND group; ^#^ *p* < 0.05 vs. Veh + HFD group.

HFD feeding for 16 weeks elevated significantly hepatic TG levels; however, this elevation was attenuated by PM extract (Figure 5A). Then, we subjected histological examination after H&E and Oil-red O staining to further investigate the anti-steatotic effect of PM extract. The liver tissues from HFD-fed mice presented predominantly macrovesicular steatosis, compared to those from ND-fed mice, which were attenuated by PM extract (Figure 5B). The PM extract also reduced accumulation of lipid droplets in the liver tissues of HFD-fed mice, as shown in Oil-red O stained images (Figure 5C).

### 3.7. PM Extract Reduced Fasting Blood Glucose Levels, Improved Glucose Tolerance, and Insulin Sensitivity in HFD-Fed Mice

**Figure 6:**
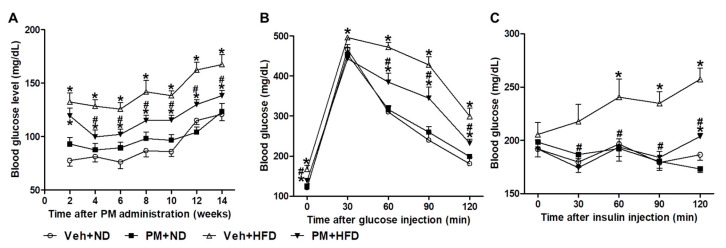
The effect of PM extract on fasting blood glucose levels, glucose tolerance, and insulin sensitivity in HFD-fed mice. The mice fed with ND or HFD were treated with PM extract or vehicle. The mice fasted for 16 h, and their blood glucose levels were measured every 2 weeks. For the glucose tolerance test (GTT), the mice fasted for 16 h before assaying. Fasting blood glucose levels (**A**), GTT performed after injecting 20% D-glucose (2 g/kg body weight) (**B**), and insulin tolerance test (ITT) performed after injecting insulin (1 U/kg body weight) (**C**). Data are the mean ± SEM. * *p* < 0.05 vs. Veh + ND group; ^#^ *p* < 0.05 vs. Veh + HFD group.

To determine the anti-diabetic effect of PM extract, fasting blood glucose levels were measured and GTT and ITT assays were performed in ND- or HFD-fed mice. Fasting blood glucose levels were measured every two weeks. HFD-fed mice presented higher fasting blood glucose levels compared to ND-fed mice; the PM extract significantly reduced the levels after two weeks of treatment in HFD-fed mice (Figure 6A). As shown in GTT and ITT assays, PM extract significantly improved glucose tolerance and insulin sensitivity in HFD-fed mice (Figure 6B,C).

### 3.8. PM Extract Modulated Lipogenic and Lipolytic Protein Levels in HFD-Fed Mice

**Figure 7:**
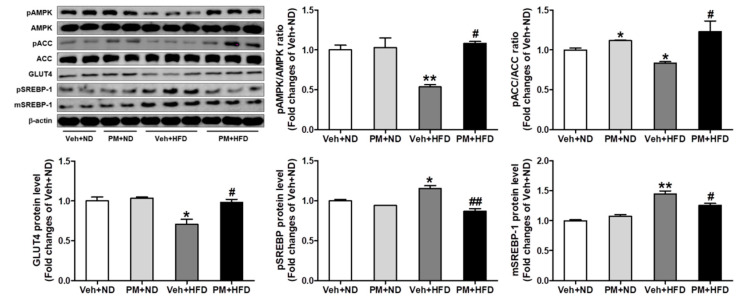
The effect of PM extract on AMPK and ACC phosphorylation, GLUT4, and SREBP-1 protein levels in the liver tissues of ND- or HFD-fed mice. Representative images of western blot analysis are shown, and densitometric quantifications of relative band intensities are presented. The mice fed with ND or HFD were treated with PM extract or vehicle for 16 weeks, and the liver tissues were collected. Data are the mean ± SEM. * *p* < 0.05 and ** *p* < 0.01 vs. Veh + ND group; ^#^ *p* < 0.05 and ^##^ *p* < 0.01 vs. Veh + HFD group.

To investigate whether PM extract affects the expression of proteins regulating lipid metabolism in HFD-fed mice, we measured the lipolytic (AMPK and ACC) and lipogenic (SREBP) proteins and GLUT4 protein levels using western blot analysis. There were no changes on total AMPK and ACC levels in all experimental groups. HFD feeding decreased the phosphorylation of AMPK and ACC and the GLUT4 protein expression, whereas it increased pSREBP and mSREBP protein levels. However, these changes were significantly attenuated by PM treatment (Figure 7).

## 4. Discussion

Here, we investigated the therapeutic effect of PM and its molecular mechanisms on the development of non-alcoholic steatosis and insulin resistance in the models of FFA-exposed HepG2 cells and HFD-fed mice.

PM, a popular herbal medicine, has had thousands of years of medicinal history in China and Asian countries treating various diseases. However, in recent years, numerous cases of PM-drug-induced liver injury (OM-DILI) have been reported, which has caused a great concern worldwide [19,20,21]. In order to remove its hepatotoxicity, we processed PM by the optimized multi-step procedures and ethanol-extraction, which was followed by filtration, evaporation, and dissolution via sonication.

We then performed the general toxicity tests of PM extract three different ways. First, in an acute toxicity test, 8-week old SD rats were administered 2000 mg/kg of PM extract five times at 1 h intervals, and exhibited no death, morbidity, or behavioral abnormality, and no changes in body weight, internal organ structure, or function. Second, in a dose-range finding study, NOAEL (no observed adverse effect level) was 1000 mg/kg for 2 weeks on SD rats. Third, in an Ames II test, up to 1000 mg/kg of PM extract did not induce reverted mutations of *Salmonella Typhimurium (His-)*. As shown in Figure 1, we also determined a non-cytotoxic concentration by MTT and RTCA assays before studying the anti-steatotic effect of PM extract in HepG2 cells.

In vitro studies clearly showed that PM extract decreased lipid accumulation in FFA-exposed HepG2 cells. Accordingly, PM extract reduced the phosphorylation of AMPK and ACC, and GLUT4 expression, as well as reduced SREBP1 expression levels. These results strongly suggest that PM extract has a direct effect on lowering lipid accumulation through AMPK and its downstream signaling. In vivo studies also showed no symptoms of hepatotoxicity by PM treatment for 16 weeks and validated the anti-steatotic effect of PM. First, PM treatment decreased the weight of adipocyte tissues and the hepatic TG and lipid accumulation in HFD-fed mice. Second, PM treatment showed a significant reduction in fasting blood glucose levels, and a significant improvement in glucose tolerance and insulin sensitivity in HFD-fed mice. Accordingly, the phosphorylation of AMPK and ACC, and GLUT4 expression were increased, and SREBP1 was decreased in the liver of PM-treated HFD-fed mice. Conclusively, PM extract alleviates non-alcoholic steatosis and insulin resistance through modulating the expression of proteins on lipid metabolism and glucose transport in the liver.

The primary source of hepatic lipids is from FFA intake from adipocyte tissue lipolysis, de novo lipogenesis, and dietary uptake; lipids are also cleared by the hepatic beta-oxidation of fatty acids, and exported as lipoprotein particles [22]. Since 60% of hepatic lipid accumulation results from FFAs of adipose tissues, the effect of PM extract on reducing excess visceral fats in HFD-fed mice correlates strongly with its anti-steatotic effect. PM extract also stimulate AMPK activation, a critical regulator of energy metabolism. The consequence of AMPK activation is stimulation of hepatic fatty acid oxidation and inhibition of cholesterol and triglyceride synthesis, inhibition of adipocyte lipolysis, stimulation of skeletal muscle fatty acid oxidation and glucose uptake, and modulation of insulin secretion by pancreatic beta-cells; thus, AMPK is an emerging drug target for diabetes and metabolic syndrome [23].

Glucose intolerance and insulin resistance are the major pathologies found in the patients of metabolic disorders [24], which were evaluated by plasma glucose and insulin levels, and GTT and ITT assays. Glucose transporters assist the transfer of glucose across membranes, and particularly, the translocation of glucose by GLUT4 is insulin-dependent, and a rate limiting step of glucose usage [25]. Up-regulated GLUT4 expression could amplify the insulin signaling and PM extract may increase glucose and insulin sensitivity in the liver of HFD-fed mice. However, it remains to be studied further how PM extract regulates the GLUT4 expression in detail. In addition, PM extract alleviated insulin resistance and hyperglycemia in HFD-fed mice. This clinical study revealed a strong association between insulin resistance and hepatic steatosis. Increased cellular fatty acids, TNFα, and AMPK have been reported to activate different stress kinases, such as insulin receptor substrate (IRS) proteins, and alter downstream kinase pathways [26]. IRS proteins have been implicated in regulating GLUT4-dependent glucose uptake in response to insulin. Moreover, many phytochemicals are reported to act on IRS-mediated insulin signaling pathways to regulate GLUT4 trafficking [27]. It remains to be studied further how PM extract regulates IRS and downstream kinases, as well as GLUT4, to improve insulin sensitivity.

Chemical constituents of PM include stilbenes, quinones, flavonoids, and other compounds; among them, stilbene glucosides and anthraquinones are two major constituents. Studies have shown that 2,3,5,4′-tetrahydroxystilbene-2-O-β-glucoside (TSG) exhibits many medicinal properties of anti-aging, cardiovascular, and neuroprotective effects [28]. On the other hand, anthraquinones possess pharmacological properties of anti-cancer, anti-inflammatory, anti-oxidant, and hepatoprotective activities [28]. Interestingly, TSG was reported to inhibit vascular remodeling and fibrosis in spontaneously hypertensive rats (SHRs) with increasing blood flow [29]. Our preliminary data also showed that PM extract increased the vascular diastolic rate of rat thoracic aorta in response to acetylcholine.

Consistent to previous reports, our HPLC results showed that the PM extract contains stilbenes, quinones, and flavonoids; among them, 2,3,5,4′-tetrahydroxystilbene-2-O-β-glucoside (TSG) and catechin were detected as main phytochemical constituents. Catechin is a natural antioxidant found in green tea, fruit, and chocolate. Many studies have shown that catechins can be effective in controlling hyperglycemia and diabetic complications by improving insulin sensitivity and reducing dyslipidemia and obesity [30,31,32]. A study also reported that catechin ameliorates adipose insulin resistance by improving oxidative stress and glucose uptake in HFD-fed obese rats and adipocytes [33]. TSG, a major component of PM, has also shown an anti-diabetic activity [34]. A recent study reported that TSG exerts a hypoglycemic effect and improved glucose and insulin sensitivity in diabetic mice [35]. Additional pharmacological studies on each component of PM may be required to understand the biological functions and possible toxicological effects of PM in the body.

A recent case-control study reported that a particular dietary pattern, called a DASH diet, correlates negatively with the development of NAFLD [36]. The DASH-style diet is rich in fruits, vegetables, whole grains, low-fat dairy products, and nuts, but is low in saturated fat, sodium, and added sugars. The other study also reported that the Western dietary pattern is prospectively associated with an increased risk of NAFLD due to high BMI, central obesity, and dyslipidemia [37]. The mechanism underlying these correlations may be through excess intake of saturated fats, sodium, and sugars. Importantly, the beneficial effects of the DASH diet are from whole grains, nuts, and vegetables because they contain phytochemical components such as phenolic acids, flavonoids, and saponins, which have been known for their antioxidant and anti-inflammatory activities [38,39,40]. Moreover, dairy products contain probiotics to improve digestion and absorption [41,42]. Thus, dietary supplements may protect from liver diseases and alleviate the pathology of NAFLD and other metabolic diseases. Here, we provided the experimental data showing that PM extract alleviates non-alcoholic steatosis and insulin resistance in HFD-fed mice.

## 5. Conclusions

In conclusion, we investigated the therapeutic effect of PM extract after the multiple processing and extraction procedures done in order to reduce hepatotoxicity. PM extract was found to modulate the AMPK-mediated lipid metabolism and decrease hepatic lipid accumulation directly. The present study strongly supports the therapeutic potential of PM extract on alleviating non-alcoholic steatosis and insulin resistance, as well as metabolic complications associated with obesity.

## Figures and Tables

**Table 1:** Phytochemical contents in the Polygoni multiflori (PM) extract.

Phytochemical	Content ^1^ (mg/g)
Catechin	1.51 ± 0.07
2,3,5,4′-tetrahydroxystilbene-2-O-α-glucoside (TSG)	36.68 ± 1.83
Rhein	0.3 ± 0.02
Emodin	0.05 ± 0.00
Chrysophenol	ND ^2^
Total	38.54 ± 1.93

^1^ All values are presented as the mean from three independent determinations. ^2^ ND, non-detectable.
